# The Effect of SiO_2_ Particle Size on Crystallization Behavior and Space Charge Properties for SiO_2_/MMT/LDPE Composites

**DOI:** 10.3390/ma17071605

**Published:** 2024-03-31

**Authors:** Hongtao Jiang, Hong Yuan, Qunguang Yu, Jing Xie

**Affiliations:** 1 Postdoctoral Scientific Research Mobile Station, Jinan University, Guangzhou 510632, China; tyuanhong@jnu.edu.cn; 2 Postdoctoral Innovation Practice Base, Guangzhou Cable Works Co., Ltd., Guangzhou 511480, China; yuqg@gzdlc.com.cn (Q.Y.); xiej@gzdlc.com.cn (J.X.); 3 School of Architectural Engineering, Guangzhou Institute of Science and Technology, Guangzhou 510540, China

**Keywords:** different dimensions, inorganic filler, multicomponent composites, crystallization behavior, electrical properties

## Abstract

The matrix material used in this paper was low-density polyethene (LDPE), and the added particles selected were silicon oxide (SiO_2_) particles and montmorillonite (MMT) particles. The sizes of the SiO_2_ particles were 1 µm, 30 nm, and 100 nm, respectively; three kinds of SiO_2_/MMT/LDPE multi-component composites were prepared based on MMT/LDPE composites doped with MMT particles. The effect of the SiO_2_ particle size on the crystallization behavior and space charge properties of SiO_2_/MMT/LDPE composites was studied. The crystalline behaviors and crystallinity of the materials were analyzed. At the same time, the changes in the relative dielectric constant ε_r_ and loss factor tanδ for each material with the influence of frequency were studied, and the space charge accumulation, residual characteristics, and apparent charge mobility of each material were explored. The results show that the smaller the size of the added particles, the smaller the grain size and the clearer the grain outline for the multi-composite material. After adding 30 nm SiO_2_ particles, the crystallinity of the material increases significantly. The microstructure formed by the addition of 100 nm SiO_2_ particles effectively restricts molecular chain movement and makes it difficult to establish the polarization of the composite. The incorporation of large-size particles can reduce the proportion of the crystalline structure for the material as a whole, resulting in the formation of a new structure to promote charge transfer. Among the three kinds of SiO_2_ particles, the addition of 30 nm SiO_2_ particles can effectively suppress the space charge, and the composite material has the lowest residual space charge after depolarization. The addition of 100 nm SiO_2_ particles can cause the accumulation of many homopolar charges near the anode.

## 1. Introduction

The high-voltage direct current (HVDC) cable is the key to flexible direct current (DC) transmission technology, which plays a vital role in long-distance transmission, cross-sea power transmission, and wind power integration [1,2,3,4]. Heretofore, more than twenty HVDC plastic cable transmission projects have been put into operation worldwide, with a total length of more than 3000 km. They play a crucial role in offshore wind power integration, island power supply, and the interconnection of synchronous/asynchronous power grids. Low-density polyethene (LDPE) is widely used in the insulation materials of HVDC cables because of its high insulation resistance, good voltage resistance, low dielectric loss, convenient processing, low cost, and good corrosion resistance. However, limited by its inherent properties, LDPE is prone to space charge accumulation under DC field strength, which in turn induces electric field distortion. Especially in the state of polarity inversion, it is easy to make the cable insulation breakdown. Therefore, the stable operation and voltage level improvement of polyethylene-insulated HVDC cables are greatly restricted [5,6,7,8].

Over the years, numerous scholars have found that the addition of inorganic particles to composites can effectively increase the breakdown field strength, suppress space charge, and even modulate carrier flow [9,10,11,12,13]. This method of improving certain physical and chemical properties by adding inorganic particles is widely used in industry and engineering. This provides a technical guarantee for high-voltage DC cable insulation production.

Montmorillonite (MMT), as a natural lamellar nanostructured silicate mineral, can be formed under pure natural conditions with abundant output. It has strong adsorption and cation exchange properties. As a filler, it can play the role of a barrier inside the material, change the migration path of small molecules, and reinforce the mechanical, thermal, and electrical performances for composites. It is often referred to as “all-purpose material” [14]. For example, Ahmed et al. modified flexible polyurethane (FPU) foam by incorporating a natural montmorillonite (Na-MMT) nanoclay, which increased the compressive strength of the material by 27.75% [15]. Ruan et al. successfully used a 1% sodium montmorillonite (MMT-Na^+^) clay modified by bis –(1-butyl-3- methylimidazole) zinc tetrachloride (bmim_2_ [ZnCl_4_]) to modify the epoxy coating, which made it have an excellent corrosion resistance and self-repairing ability. [16].

The use of different particles to fill polymers can make the material exhibit excellent performance in one or several aspects, such as acoustics, optics, heat, electricity, and mechanics. Moreover, due to the different types, numbers, and surface thickness of functional groups in different particles, when particles are bonded to the matrix material, particles will be attracted to each other, thereby showing synergistic effects [17]. For example, Kaffayatullah et al. produced high-performance concrete by adding a lot of basaltic volcanic ash, metakaolin (MK), micro-silica (MS), and nano-silica (NS). Binary mixtures, including fine (VA) and ultrafine (VAF) pozzolans, and ternary mixtures, including the combination of VA and MK and MS and NS, were prepared. They found the ternary mixture has good strength and high resistance to chloride ion penetration and water absorption. All binary and ternary mixtures showed low autogenous shrinkage and low drying shrinkage [18]. Imai et al. used SiO_2_ and layered silicate as micro- and nano-additive particles, based on epoxy resin. In their research, it was found that co-doping micro-nanoparticles can lead to optimal pressure resistance and dielectric strength [19]. In literature [20], we added SiO_2_ and MMT particles to LDPE to improve its electrical properties. We focused on the effect of particle addition order on the properties of composites. Considering that the size of the added particles is also an essential factor affecting the properties of materials, continuous research has been carried out in this paper.

In this paper, 1 μm, 30 nm, and 100 nm SiO_2_ particles were selected and co-doped with MMT particles into a LDPE matrix to prepare three kinds of multi-component composites. The effect of SiO_2_ particle size on crystallization behavior and space charge properties for SiO_2_/MMT/LDPE composites was studied. The change in crystallization behavior for multi-component composites caused by the addition of particles with different sizes was researched. The effects of the microcrystal structure change on macroscopic dielectric properties, space charge accumulation properties, and apparent charge mobility for the composites were discussed. Finally, the relationship between the microstructure and macroscopic electrical properties was explored. This is instructive for building a bridge between the microstructure and macroscopic electrical performances of materials.

## 2. Experimental Methods

### 2.1. Experimental Materials and Samples

The MMT particles were provided by Qinghe Chemical Plant (Zhangjiakou, China). The particle size range is from 40 to 70 μm. An intercalating agent and octadecyl trimethyl ammonium chloride was provided by Sinopharm Chemical Reagent Co., Ltd. (Shanghai, China). The 1 µm, 30 nm, and 100 nm SiO_2_ particles were supplied by Beijing Deke Daojin Science and Technology (Beijing, China). LDPE was supplied by Jinshan Petrochemical Company (Shanghai, China).

Firstly, 20 g MMT was dissolved in a glacial acetic acid aqueous solution with a pH of 3.5. Under 80 °C water bath heating, the mixed solution was evenly stirred for 1 h with a motor. We obtained the suspension. The suspension was centrifuged and purified, and then an octadecyl trimethyl ammonium chloride solution with an organic cation content of 36 mmol was added. The above suspension was stirred and ultrasonically treated for 2 h under heating in a water bath. The intercalation of MMT was completed. The cavitation energy of ultrasonic action will continuously make the MMT particles vibrate and collide, which can make them get rid of the interlayer restraint and realize secondary exfoliation [21,22,23]. After this, the static suspension was washed with demineralized water until no white precipitation appeared when detested with 1% AgNO_3_ solution. Finally, the suspension was dried, milled, and sieved to obtain the solid MMT particles required for the experiments [24]. LDPE was used as the matrix, and MMT particles and SiO_2_ particles of 1 μm, 30 nm, and 100 nm were used as additives. All kinds of particles were mixed with LDPE by adding MMT particles first and then adding SiO_2_ particles. The total mass of the LDPE and particles used in each composite material was 40 g, and all the particles were added according to the mass fraction of 1%. Particles and matrix materials were mixed in a melt-blending manner by a torque rheometer. Four different SiO_2_/MMT/LDPE composites were prepared. The mixing temperature was set to 140 °C, the screw speed was set to 40 r/min, and the processing time was 20 min. Then, each material was treated by a flat vulcanization mechanism. The temperature was set at 140 °C. The gradient boosting method was used to increase the pressure for a total of 15 min. Finally, the samples required for experiments was obtained after water cooling. The composition information for each composite material is shown in Table 1.

### 2.2. Instrumentation and Equipment

The RM-200A torque rheometer (Hapu Electrical Technology Limited Liability Company, Harbin, China), Leica DM2500 polarizing microscope (PLM, Leica Microsystems, Wetzlar, Germany), DSC-1 differential scanning calorimeter (DSC, Mettler Toledo, Zurich, Switzerland), broadband dielectric/impedance spectrometer (Novocontrol Technologies, Montabaur, Germany), and pulsed electro-acoustic space charge test system (Shanghai Jiao Tong University, Shanghai, China) were used for the experiments.

### 2.3. Crystallization Behavior

A mixed solution of potassium permanganate (KMnO_4_) and concentrated sulfuric acid (H_2_SO_4_) with a mass fraction of 5% was used as the etching solution. The experimental materials were etched for 4 h, and the solution was agitated each 30 min. After etching, each material was washed with deionized water and cleaned with an ultrasonic wave. Then, all samples were observed and photographed under the polarizing microscope.

Specimens were tested by the DSC-1 differential scanning calorimeter. The rising and cooling rate was 10 °C/min, and the temperature range was 25~150 °C. The amount for each specimen was 10~15 mg, and the whole process was carried out under N_2_ atmosphere. The specimens were raised from 25 to 150 °C and then cooled down to 25 °C. This is to remove the thermal history of the various polymers themselves so that polymers melt and become a homogeneous distribution of molten states. The ordered structure within the material was eliminated and became an utterly disordered melt. Afterwards, the temperature slowly increased to 150 °C, and the differential scanning calorimetry (DSC) curves were plotted.

### 2.4. Dielectric Frequency Spectra

The variation of ε_r_ and tanδ with frequency *f* for every specimen was tested by a broadband dielectric/impedance spectrometer at ambient temperature (25 °C). The frequency range was selected from 10^−1^ to 10^6^ Hz. It is worth noting that to decrease the effect of moisture and residual charge on results, each material needs to be short-circuited for 24 h in advance. The diameter of the material used in the experiment was 35 mm, and the thickness was 200 μm. Aluminum electrodes with a diameter of 25 mm were plated on both sides of the material.

### 2.5. Space Charge

The space charge characteristics for various materials were measured by the space charge experimental device in the pulse electroacoustic (PEA) method. The rationale is that a nanosecond high-voltage narrow pulse wave is injected into the material by electrode. And the propagation of the high-voltage narrow pulse wave inside the materials will format different perturbations to the various bound charges, which will cause the bound charges to have different extents of small displacements. Then, the sound wave propagates to the opposite electrode. The polyvinylidene fluoride (PVDF) piezoelectric sensor is used to collect and process the acoustic signal, which is converted into the corresponding electric pulse signal. The computer software processes and analyzes the electric pulse signal to obtain the space charge distribution at different locations within materials.

**Figure 1:**
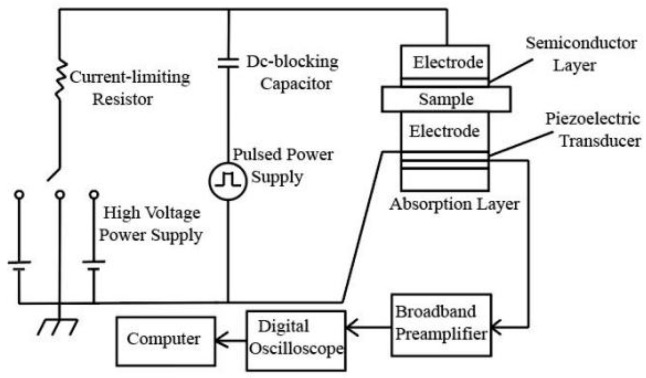
Structure for space charge test device.

The specific test device structure is shown in Figure 1. Specifically, it includes a 0~20 kV DC power supply, a pulse generator with the maximum pulse of 1.0 kV and width of 15 ns, a 30 μm thick PVDF piezoelectric sensor, as well as a preamplifier, an oscilloscope, and a computer operation module. The PEA test system was used to test the space charge distribution characteristic curves in all composites polarized for 30 min at 10 kV/mm, 20 kV/mm, and 30 kV/mm field strengths. The depolarization space charge distribution curves for all materials were tested after 30 min of short-circuiting.

## 3. Results and Discussion

### 3.1. Crystalline Morphology of Composites

**Figure 2:**
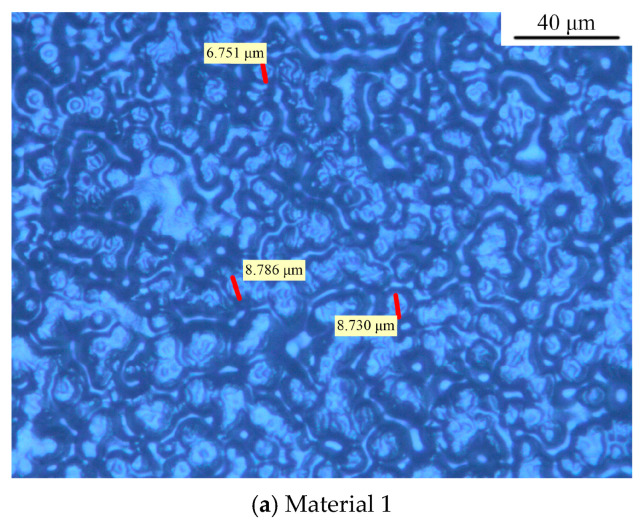

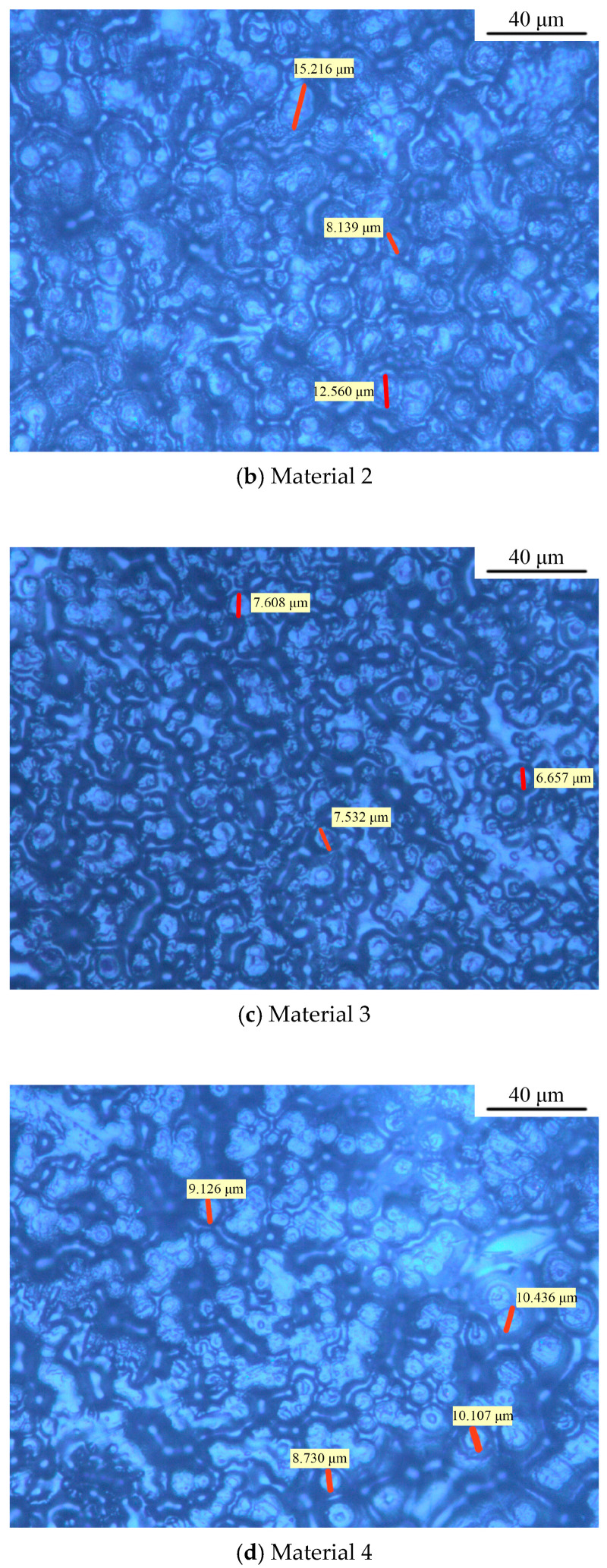
The crystalline morphology for each material.

**Figure 3:**
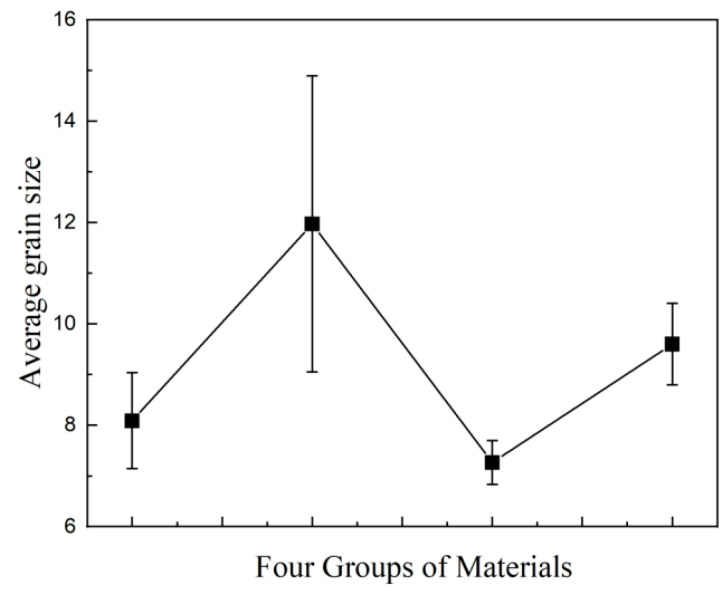
Statistical distribution of grain size for each material.

MMT, 1 μm SiO_2_, 30 nm SiO_2_, and 100 nm SiO_2_ particles were mixed with LDPE using a melt-blending technique. The crystalline morphology for each material after etching, observed under the polarizing microscope, is shown in Figure 2. As can be seen, the grains for four materials are all spherical structures. Based on the grain size data in Figure 2, the statistical distribution of grain sizes for each material was calculated. The results are shown in Figure 3.

In Figure 2 and Figure 3, the grain diameter of material 1, doped only with MMT particles, is about 6 to 9 μm, and the average grain size is around 8.09 μm. After SiO_2_ particles were doped in material 1, due to the addition of two kinds of particles, many particles play the role of heterogeneous nucleation, forming a compact crystal structure, and the grain outline is clear.

Three kinds of composite materials containing SiO_2_ particles were compared. The grain size of material 2 increases after adding 1 μm SiO_2_ particles, but the dispersion is large, and the average grain size is about 11.97 μm. The grain size of material 3 decreases after the addition of 30 nm SiO_2_ particles. The mean value of its diameter is about 7.26 μm, and the scale is relatively uniform. The distinction between the crystalline region and amorphous region is obvious. The average grain size of material 4 is 9.59 nm after doping with 100 nm SiO_2_ particles. This value is between material 1 and material 2, and the grain spacing is small. However, the boundary between the crystalline and amorphous regions is slightly blurred compared to material 3.

### 3.2. DSC Testing of Composites

**Figure 4:**
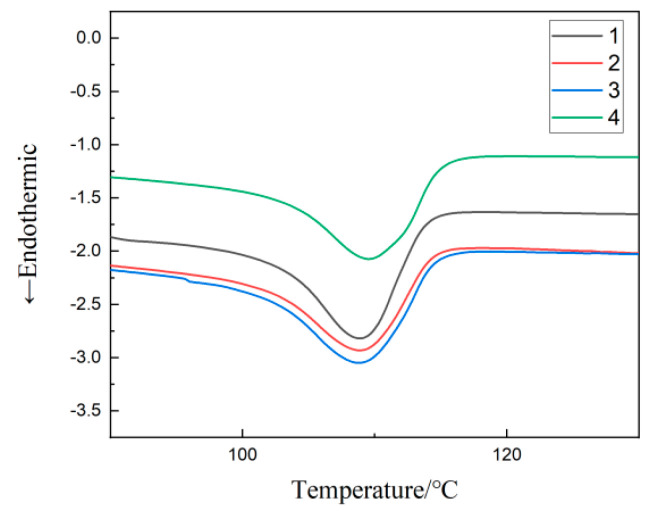
DSC curves for each material.

The DSC test was applied to four materials, and the results are shown in Figure 4. The melting peak temperature of each composite material can be acquired from the equipment, as shown in Table 2. The melting enthalpy $∆H_{m}$ for every material is calculated by Formula (1) [25]:

(1) $$∆H_{m}=60\int_{T_{i}}^{T_{f}}\frac{Q_{H(T)}}{B}dT$$

where $T_{i}$ and $T_{f}$ imply the values of the initial and ending temperatures of the characteristic peaks for the materials melted. $Q_{H(T)}$ represents the rate of increase and decrease for the heat flux, and the unit is ${W\cdot g}^{-1}$. B represents the rate for the temperature rise and fall. The calculated results of $∆H_{m}$ are shown in Table 2.

The specific values of crystallinity for four materials are calculated by Formula (2) [26]:

(2) $$X_{c}=\frac{{∆H}_{m}}{(1-w){∆H}_{m}}\times 100\%$$

where $H_{0}=293.6\;J\cdot g^{-1}\;$represents the crystallization melting enthalpy for LDPE and w represents the mass percentage of inorganic particles in the composites. The calculated results of $X_{c}$ are also shown in Table 2.

By comprehensively comparing Figure 4 with Table 2, it is possible to determine the ordering of melting temperatures for the four materials: material 4 > material 1 > material 3 > material 2. The order of crystallinity is as follows: material 3 > material 4 > material 1 > material 2. Among the three kinds of composites containing nanoparticles, the crystallinity of material 3 with 30 nm SiO_2_ particles is the highest. This shows that the addition of small-sized particles plays a good role in heterogeneous nucleation. The addition of 1 μm SiO_2_ particles and 100 nmSiO_2_ particles, due to the large particle size, increases the restriction of the molecular chain movement in materials, further hindering the crystallization process. Therefore, the crystallinity of material 2 and material 4 is lower than material 3.

### 3.3. Dielectric Spectrum of Composites

**Figure 5:**
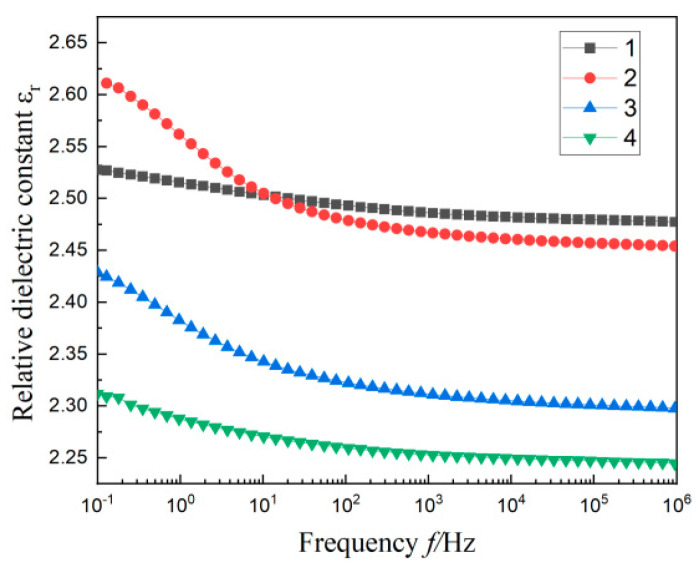
The variation curves of ε_r_ with *f* of each composite material.

**Figure 6:**
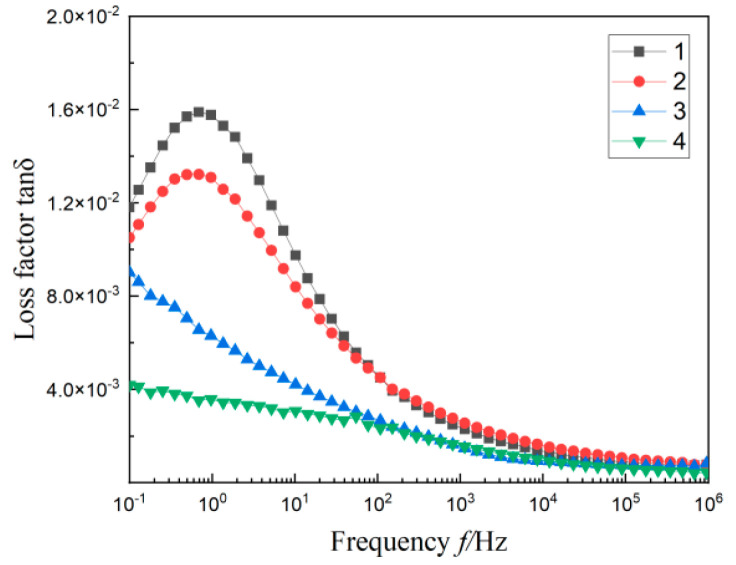
The variation curves of tanδ with *f* of each composite material.

The test results of the relative permittivity ε_r_ and loss factor tanδ for four materials with frequency *f* are shown in Figure 5 and Figure 6. It is not difficult to find that the dielectric constant of material 1 is distributed between 2.48 and 2.52, and there is an apparent dielectric loss peak in the lower frequency band (0.1~10 Hz). This is due to the different limiting effects of MMT particles on the intercalation agent, resulting in functional group orientation polarization and Maxwell–Wagner polarization [27,28].

According to the PLM diagram, the heterogeneous nucleation caused by the addition of two particles will form a compact crystalline structure in the matrix. The closely arranged structure will limit the movement of molecular chains, which will make it difficult to establish polarization. Therefore, after adding SiO_2_ particles of different sizes in material 1, the dielectric constant and loss of material 2, material 3, and material 4 are decreased to various degrees.

From Figure 5, in the lower frequency band (0.1~10 Hz), the dielectric constants of three materials containing SiO_2_ particles are significantly improved compared with the whole frequency band (0.1~10^6^ Hz). Under the action of an external field, the interface structure formed by SiO_2_ particles in the matrix will be equivalent to large dipoles when they contact each other. When the frequency of the electric field is low, the charge in the dielectric bilayer moves directionally and triggers dielectric relaxation, resulting in the “quasi-DC” phenomenon [29]. Therefore, the dielectric constant transition of the three materials gradually increases with the decrease of frequency at a low frequency.

According to the experimental data in Figure 4 and Table 2, the multi-component polymer molecules with 1 μm SiO_2_ particles have low crystallinity and a small proportion of crystalline regions. This provides sufficient space for the movement of molecular chains. There is obvious interfacial relaxation polarization between the inorganic particles and polymer matrix. These result in the relative permittivity and loss of material 2, which is the largest among the three multi-composites containing SiO_2_ particles. Materials with the added SiO_2_ nanoscale particles, both 30 nm and 100 nm, have small grain sizes and a tight arrangement. This leads to their relatively poor molecular chain activity and difficulty establishing polarization. Therefore, their relative permittivity is small. For the multi-composites with 100 nm SiO_2_ particles added, the size of the added particles are smaller than 1 μm SiO_2_ particles. Compared with material 1, its crystal area accounts for a relatively large proportion, and the grain distribution is relatively close. This arrangement may make the movement of molecular chains limited, resulting in a small ε_r_ value and a low tanδ value of material 4 in Figure 5 and Figure 6. The small size 30 nm SiO_2_ particles added in MMT/LDPE can form a small size and small spacing grains. According to Reference [30], these particles with very small spacing will form tiny capacitors. Therefore, the dielectric constant and dielectric loss of material 3 are slightly higher than material 4; the results shown in Figure 5 and Figure 6.

### 3.4. Space Charge Characteristics of Composites

**Figure 7:**
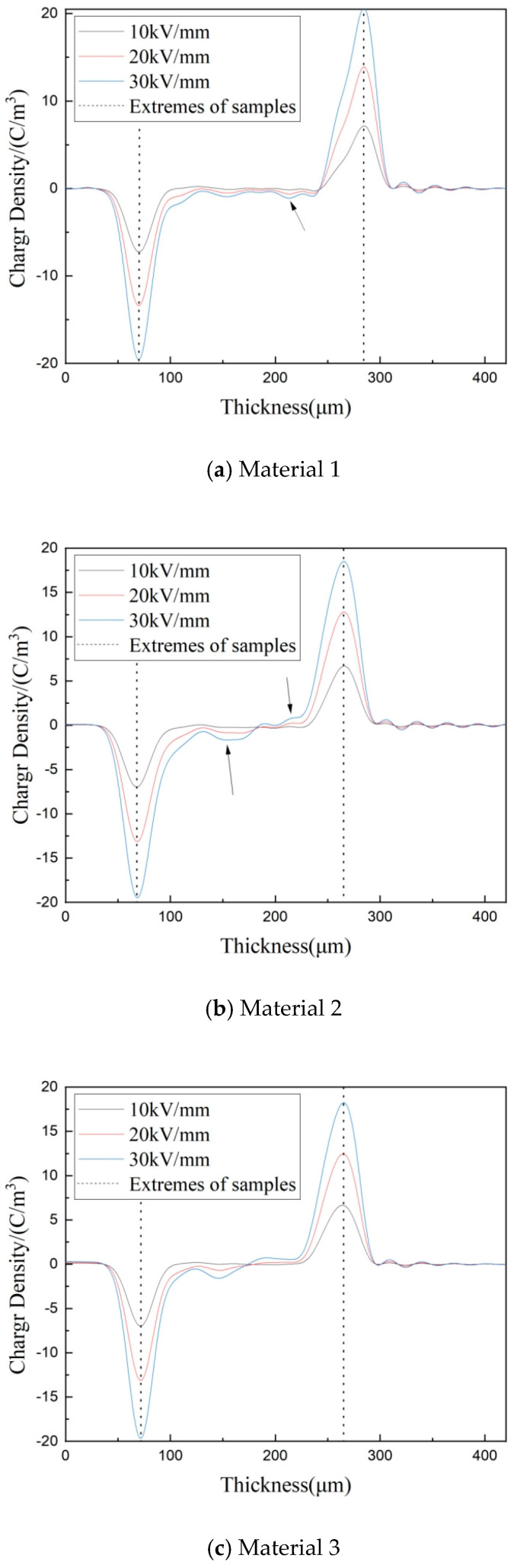

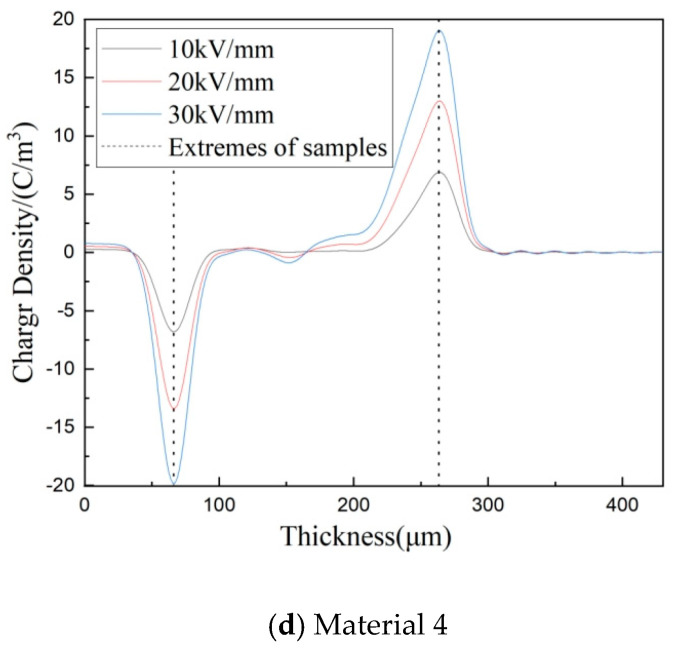
Space charge distribution curve of each composite material.

Figure 7 shows the test results of the internal space charge distribution for material 1, material 2, material 3, and material 4 after polarization at 10 kV/mm, 20 kV/mm, and 30 kV/mm DC field strength for 30 min, respectively. The thickness of each test material is 200 μm. The positions of the negative and the positive electrodes were marked with dotted lines in the figure. When the external electric field acts on the material, the charges released by the electrode will be captured by the traps inside the material. This will accumulate homopolar charges near the electrode and weaken the field strength. At the same time, there is an interface polarization between the electrode and the medium, which will produce dipole charges. Or the heteropolar charge is generated due to the ionization of impurities inside the material. When these heteropolar charges move in the opposite direction of the electric field, they will change the space charge distribution in the medium through a series of processes such as trapping, de-trapping, or compounding with homopolar charges [20].

The matrix material of each composite, polyethene, is a typical semi-crystalline polymer. Its molecular structure and morphology are associated with charge injection, transport, and trapping. And polyethene is composed of two parts: the crystalline region and the amorphous region. Residual free volume, double bonds, end groups, and interfaces between crystalline and amorphous regions all give rise to new localized states. These newly born localized states can act as traps, capturing and hindering the charge migration, and then form a space charge.

From Figure 7a, material 1 has almost no obvious charge accumulation under the 10 kV/mm field strength. As the field strength rises, the negatively polarized charge aggregation in the middle of the material gradually increases. When the field strength rises to 30 kV/mm, the maximum accumulation of the charge inside material 1 is 1.15 C/m^3^, which is the position of the arrow in the figure. Although the addition of MMT particles can eliminate some of the localized defects within the matrix material through heterogeneous nucleation, it can also result in the formation of new free volume, end groups, etc. This generates new localized states in the material, causing charge traps that trap and impede charge migration. This caused the accumulation of a space charge inside the material and will not completely disappear.

After adding 1 μm SiO_2_ particles, the space charge accumulation in material 2 increases. The interleaving of positive and negative polarity charges occurs in Figure 7b. The maximum charge accumulation near the cathode is 1.61 C/m^3^, and the maximum charge accumulation near the anode is 1.04 C/m^3^, which are the position of arrows in the figure. From the analysis results of the PLM and DSC tests, the addition of particles changes the crystallization behavior in composites, and the grain size of material 2 increases and the crystallinity decreases, which will generate many structural defects and increase the number of traps. At the same time, the decrease in crystallinity will also reduce the scattering effect of grains on the charges and reduce the neutralization probability of positive and negative charges. Moreover, the interface between the inorganic micron particles and polymer matrix has a weak binding zone, which will lead to new local states in the material. All these lead to the accumulation of a space charge inside material 2, hence the result in Figure 7b.

Compared with material 1, material 3, which has 30 nm SiO_2_ particles, has a higher crystallinity, smaller grain size, and more uniform grain distribution, creating more firm interfaces in the material. At the same time, due to the scattering effect of the interface between the crystalline region and the amorphous region, as well as between particles, the obstacles to charge migration are increased. This is beneficial to the neutralization of positive and negative charges. Therefore, the space charge accumulation of material 3 is small.

According to the previous analysis of this paper, after adding 100 nm SiO_2_ particles, 100 nm SiO_2_/MMT/LDPE composites will form complex local state structures and introduce many traps. As seen from Table 2 and Figure 2, the crystallinity of material 4 is smaller than material 3 and larger than material 2, and the grain size is smaller than material 3. Therefore, this shows that the internal space charge accumulation of material 4 in Figure 7d is smaller than material 1, but the charge accumulation near the anode is obviously larger than material 3.

### 3.5. Depolarization Space Charge Characteristics of Composites

**Figure 8:**
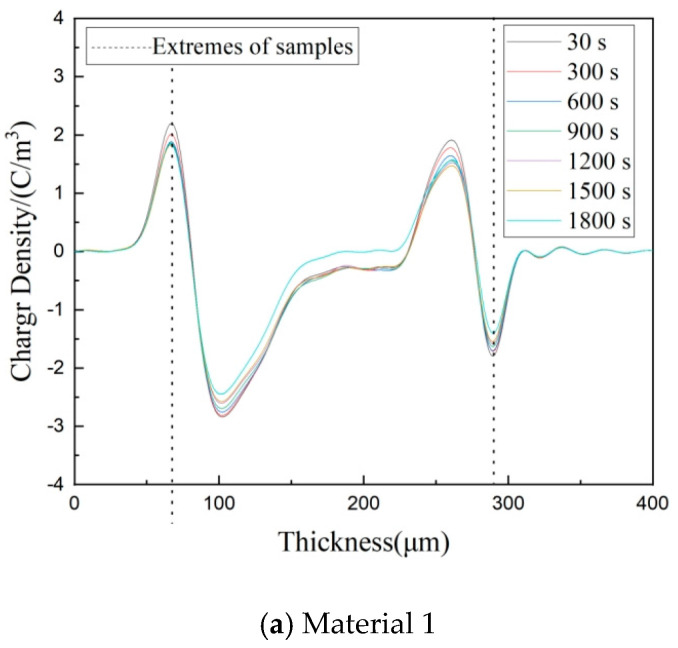

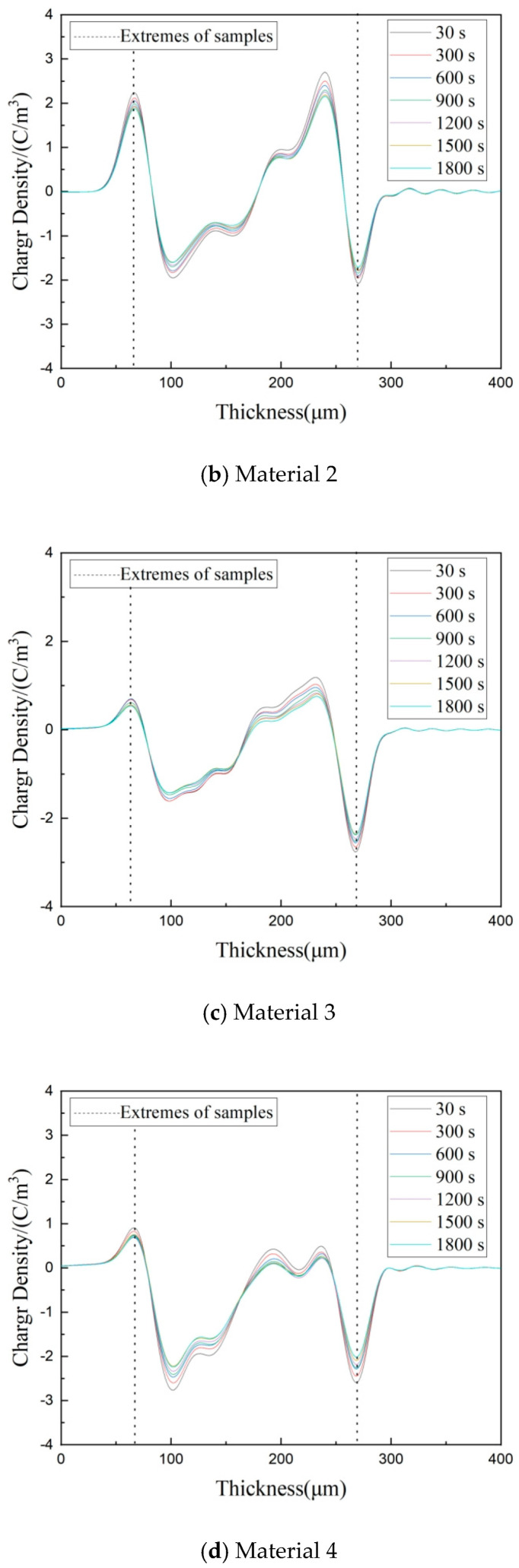
Residual space charge distribution curve of each composite material.

Figure 8 shows the space charge distribution of each composite when the applied electric field is removed and short-circuited for 30 min. The positions of the negative and positive electrodes were marked with dotted lines in the figure. The data obtained in the initial stage of the short circuit fluctuates greatly due to the limitations of the signal acquisition system. The short circuit space charge analysis in this paper is calculated from 30 s. From Figure 8, at the initial stage of the short circuit, material 1 accumulates a lot of negative charges near the electrode, the maximum residual charge near the cathode is 2.84 C/m^3^, and the maximum residual charge near the anode is 1.93 C/m^3^. In composites containing SiO_2_ particles, the residual amount of space charge for material 2 is the largest. The maximum residual charge near the anode and cathode is 1.95 C/m^3^ and 2.78 C/m^3^, respectively. The depolarization space charge curves for material 3 and material 4 have similar trends. The maximum residual charge near the anode and cathode of material 3 is 1.64 C/m^3^ and 1.19 C/m^3^, while material 4 is 2.71 C/m^3^ and 0.52 C/m^3^.

The residual space charge density of the different samples during depolarization was calculated according to Formula (3) to further analyze the influence of three scale SiO_2_ particles on the space charge accumulation of MMT/LDPE composites [31,32]. The expression of Formula (3) is as follows:

(3) $$Q\left(t,E_{p}\right)=\frac{1}{x_{1}-x_{2}}\int_{x_{0}}^{x_{1}}|q_{p}\left(x,t,E_{p}\right)|dx$$

where $q_{p}\left(x,t,E_{p}\right)\;$ is the charge density inside each material, *t* is the time during which voltage is applied, and *Ep* is the electric field intensity of 30 kV/mm. $x_{0}$ and $x_{1}\;$ are each taken at the position of critical 0 (*y* = 0) on the charge between the lower electrode and the upper electrode, respectively, to minimize the effect of induced charges at the electrodes.

**Figure 9:**
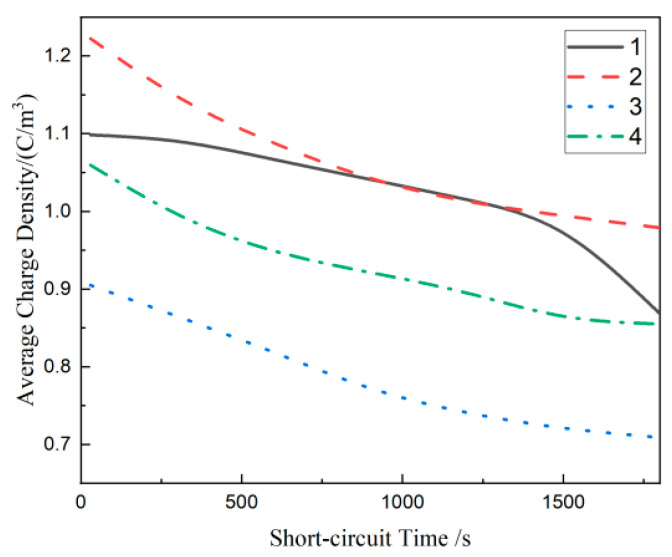
Average charge density distribution curve of each composite material.

The residual mean charge densities of material 1, material 2, material 3, and material 4 were calculated separately to obtain the distribution curves of the residual mean charge density during the depolarization of each material, as shown in Figure 9. The average charge density of each material decreases exponentially with the increase of time and finally tends to become stable gradually. After adding SiO_2_ particles, the curves of the three materials changed compared with material 1. According to the analysis in Figure 5, the addition of SiO_2_ particles can form a dielectric double-layer structure in the matrix material. The charge is easy to migrate in this structure. Hence, the residual charge density variation curves with time of material 2, material 3, and material 4 is different from material 1. The average charge density of material 3 and material 4 is lower than material 1, while material 2 is significantly higher than material 1.

Apparent mobility was used as a means of obtaining specific charge mobility values based on average charge density curves by Mazzanti G et al. [33,34,35]. Shallow traps inside the material easily capture and release the charge, while deep traps capture the charge, and the charge is difficult to escape. Therefore, in the short-circuit process, the charge limited by the shallow trap releases fast, and the charge trapped in the deep-level trap releases slowly. The expression of the calculation Formula (4) of the apparent charge mobility $\mu_{t}$ is as follows:

(4) $$\mu_{t}=\frac{\varepsilon_{0}\varepsilon_{r}}{{q(t)}^{2}}|\frac{dq\left(t\right)}{dt}|$$

where $\frac{dq(t)}{dt}$ is the slope of the average charge density after short-circuiting, and its numerical size reflects the fast and slow decay of charge, so the absolute value is taken in the formula. $q\left(t\right)\;$ is the instantaneous value of the average charge density. $\varepsilon_{r}$ and $\varepsilon_{0}\;$are the relative permittivity and vacuum permittivity, respectively.

**Figure 10:**
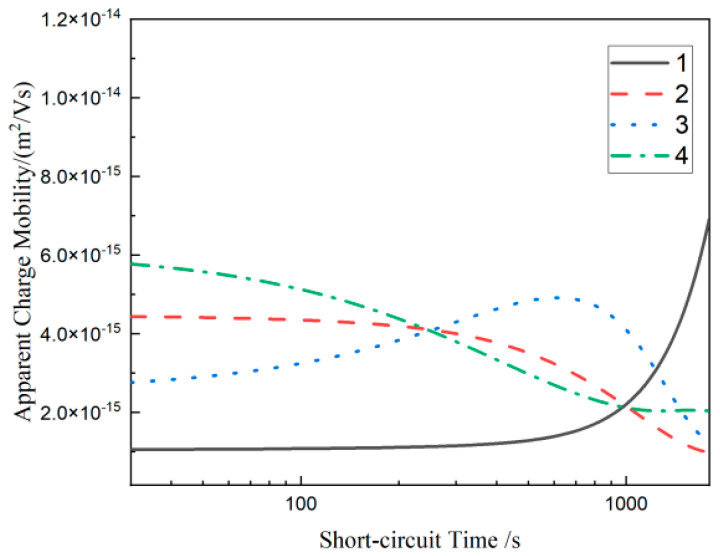
Apparent charge mobility curves of each composite material.

The calculation results of the apparent charge mobility curves of four materials are shown in Figure 10. At the beginning of the short circuit (30~900 s), the apparent mobility of the three composites containing SiO_2_ particles is significantly higher than material 1. Compared with PLM data, this is because the grain growth of multi-component composites is compact and regular after adding SiO_2_ particles, which makes the internal traps shallow. At the late stage of the short circuit (900~1800 s), the apparent mobility of material 1 reaches the maximum value among the four materials. Combined with the average charge density distribution curve in Figure 8, the residual charge density of material 1 is high. This suggests that there are many shallow traps within material 1. Shallow traps provide a transition channel for the movement of charges, and the apparent charge mobility increases obviously [36].

After adding 1 μm SiO_2_ particles, the apparent mobility of material 2 is at a high level at the initial stage of the short circuit (30~300 s). This shows that there are a lot of shallow traps in material 2, which is consistent with the analysis results of the DSC testing, dielectric spectrum, and space charge accumulation. In the later period of the short circuit (300~1800 s), the apparent mobility of material 1 decreases. This shows that the addition of 1 μm SiO_2_ particles introduces deep trap energy levels to some extent, but these traps are not enough to promote the neutralization of positive and negative charges, thus forming a lot of charge accumulation.

After adding 30 nm SiO_2_ particles, the apparent mobility of material 3 is at a low level at the initial stage of the short circuit (30~300 s). This shows that the addition of 30 nm SiO_2_ particles introduces deep traps in the material. These traps can form charge accumulation with the same polarity after capturing charges, especially in a high electric field, as shown in Figure 7c [37]. This can create an interfacial counter electric field at the electrode–sample interface, inhibiting the further injection of electrons or holes. In the later period of the short circuit (300~1800 s), the apparent mobility of material 3 increases. This indicates that the compact crystalline structure formed by the addition of 30 nm SiO_2_ particles makes some of the trap energy levels in the materials shallow, which promotes the charge migration even more. The space charge accumulation of material 3 is the lowest among the three multicomposites due to the combination of multiple factors.

After adding 100 nm SiO_2_ particles, the internal local state structure of material 4 becomes complex, and many traps are introduced. From Figure 10, the apparent mobility of material 4 is the highest among the four materials at the initial stage of the short circuit, and it is almost the lowest among all materials at the later stage of the short circuit. Combined with Figure 7 and Figure 9, material 4 has greater space charge accumulation and residue, but obvious heteropolar charge accumulation is produced near the electrode, especially near the anode, compared with material 3. To some extent, this shows that the addition of 100 nm SiO_2_ particles can make some trap levels shallow but also introduce some deep traps. And these traps do not all exist in the form of composite centers, so the results of Figure 7d appear.

## 4. Conclusions

Adding SiO_2_ particles with different sizes into MMT/LDPE can make the grain profile of composites clear. The grain size decreases with the decrease of the particle size added. And the smaller the particle size of the filler, the greater the crystallinity of the material. The grain size of 30 nm SiO_2_/MMT/LDPE is the smallest among all materials, and the heterogeneous nucleation effect of the particles is obvious. In contrast, the grain size of 1 µm SiO_2_/MMT/LDPE is the largest among all materials, and the grain size varies greatly.

When adding large-size micron SiO_2_ particles into MMT/LDPE, the proportion of the crystal structure in the whole material is reduced. Further, a weak interfacial zone is formed between the inorganic particles and the polymer matrix, which leads to the increase of interfacial relaxation polarization and loss. Whereas when small-sized nano-SiO_2_ particles are added, the grain scale of the composites is fine and uniform. The proportion of the crystalline region of the material increases, and the interface between the inorganic nano-SiO_2_ particles and the polymer matrix is well bonded. Therefore, the relaxation polarization and loss of the material are reduced.

The addition of SiO_2_ particles with different sizes can change the original crystalline structure of MMT/LDPE and form a new crystalline structure and trap energy level. Among all the materials, the crystalline structure of 30 nm SiO_2_/MMT/LDPE is the most compact, the crystallinity is the highest, and the residual space charge after depolarization is the lowest. In contrast, 1 µm SiO_2_/MMT/LDPE and 100 nm SiO_2_/MMT/LDPE have certain local state defects on the crystal structure, so there are many residual space charges after depolarization.

## Figures and Tables

**Table 1:** Components for composites.

Materials	Percentage Contents/%				
	LDPE	MMT	1 μmSiO_2_	30 nmSiO_2_	100 nmSiO_2_
1	99	1	0	0	0
2	98	1	1	0	0
3	98	1	0	1	0
4	98	1	0	0	1

**Table 2:** The melting peak temperature and crystallinity for each composite.

Materials	$\mathrm{Melting}\;\mathrm{Peak}\;\mathrm{Temperature}\;T_{m}/$°C	$\mathrm{Crystallinity}\;X_{c}$/%	Melting Heat $/J\cdot g^{-1}$
1	109.58	32.45	94.33
2	107.77	28.88	83.09
3	108.33	34.42	99.03
4	110.84	33.35	95.95

## Data Availability

The data presented in this study are available from the corresponding author upon request.
